# Editorial

**Published:** 1870-07

**Authors:** 


### Editorial.

Proceedings of Societies.—In our preceding number, it was stated, that we should publish in this, the record of proceedings of the Illinois State Medical Society, and of the American Medical Association. But the space occupied by the proceedings of the Association of Medical Editors, and of the Indiana State Medical Society, in this number, necessitates a further delay. In the meantime, Dr. Atkinson, the Permanent-Secretary of the American Medical Association, has published, in pamphlet form, a full and correct record of the proceedings of the recent meeting of that organization; and all who desire a copy can obtain it, by sending their address and twenty-five cents to W. B. Atkinson, 1400 Pine Street, Philadelphia.

The following are among the more important items of business, transacted in the general sessions of the Association:—

The Committee on a National Medical College, of which Prof. F. G. Smith, of Philadelphia, was Chairman, reported progress, and the Committee was continued.

Dr. Thos. Antisell, of Washington, read a report on Veterinary Surgery, which was referred to a special committee, consisting of Drs. S. D. Gross, of Philadelphia; Thos. Antisell, of Washington; and Alex. W. Stein, of New York.

Dr. J. S. Moore, of St. Louis, offered a resolution, recommending a minimum fee, below which medical colleges should not be allowed to charge, for a regular lecture term, which was discussed and laid on the table.

The following committee was appointed to prepare a nomenclature of disease, to report next year.

*Committee on Nomenclature of Diseases.*—Drs. Francis G. Smith, of Philadelphia (Chairman); George B. Wood, of Philadelphia; S. H. Dickson, of Philadelphia; Alfred Stillé, of Philadelphia; S. E. Chaille, of Louisiana; J. J. Woodward, United States Army; G. A. Otis, United States Army; W. S. W. Ruschenberger, United States Navy; Ninian Pinkney, United States Navy; Alonzo Clark, of New York; Austin Flint, of New York; Edward Jarvis, of Massachusetts; Wm. M. McPheeters, of Missouri; L. P. Yandell, of Kentucky; A. B. Palmer, of Michigan; Theo. Parvin, of Indiana; R. F. Michel, of Alabama.

The following resolutions were offered by Dr. A. W. Stein, of New York, and adopted:—

*Whereas*, We regard the cultivation of veterinary science of the most vital importance, not only to the advancement of human medicine, but also for reasons of political economy and agricultural interests—

*Resolved*, 1. That we recommend the State and county medical societies to use their influence in the establishment and support of veterinary schools in their respective States.

2. That they ask the Governors of their respective States to recommend in their messages to their Legislatures the importance of establishing veterinary colleges, and that appropriations be made to support them.

3. That they recommend the Governors and State Legislatures when organizing Boards of Health, to appoint one or more thoroughly educated veterinary surgeons to be associated as commissioners with the other officers of the Board.

Drs. Joseph Parrish, Willard Parker, and W. H. Mussey were appointed a committee, to report on the feasibility of establishing institutions for the cure of persons addicted to the use of intoxicants, and on the most approved methods of conducting such institutions.

The following resolutions were adopted:—

On motion of Dr. B. F. Dawson, of New York, it was

*Resolved*, That as the medical press of this country is recognized as holding a position of great power and high character,

Be it *resolved*, That in order to still further elevate its character and augment its influence, the editors of the medical Journals of this country be requested by this Association to refuse to notice, in their journals, all patent medicines, instruments, works of unprofessional or unscientific character, non-chartered institutions or hospitals, and the cards of specialists. And that advertisements of the above be refused insertion in their respective journals. And further, that such journals declining to obey the above be refused the courtesy of exchange, and their editors be refused membership in the Association of American Medical Editors.

The following officers were chosen for the ensuing year:—

PRESIDENT—Dr. Alfred Stillé, of Pennsylvania.

VICE-PRESIDENTS—1. Dr. J. S. Wetherly, of Alabama; 2. Dr. Henry Gibbons, of California; 3. Dr. T. J. Heard, of Texas; 4. Dr. Samuel Willey, of Minnesota.

ASSISTANT-SECRETARY—Dr. Joseph C. Tucker, of California.

TREASURER—Dr. Caspar Wister, of Pennsylvania.

LIBRARIAN—Dr. F. A. Ashford, of District of Columbia.

*Committee of Arrangements.*—Chairman, Drs. A. N. Sawyer; Brown, U.S.N.; J. C. Tucker, Shurtleff, Holman, Murray, (U.S.A.), and Simmons, of California; Horace Carpenter, of Oregon; Bronson, of Nevada.

*Committee of Publication.*—Drs. F. G. Smith, of Pennsylvania, Chairman; Wm. B. Atkinson, of Pennsylvania; J. C. Tucker, of California; F. A. Ashford, of District of Columbia; Căspar Wister, of Pennsylvania; H. F. Askew, of Delaware; James Aitken Meigs, of Pennsylvania.

*Committee on Prize Essays.*—Drs. T. M. Logan, of California (Chairman); H. Gibbons, H. H. Toland, Beverly Cole, Cooper Lane, of California.

Place of meeting, San Francisco, California.

Time of meeting, 1st Tuesday in May, 1871, at 11 A.M.

SECTIONS.—*Chemistry and Materia Medica.*—Drs. D. W. Yandell, of Kentucky (Chairman); H. S. Hurd, of Illinois (Secretary).

*Practical Medicine and Obstetrics.*—Drs. H. R. Storer, of Massachusetts (Chairman); J. K. Bartlett, of Wisconsin (Secretary).

*Surgery.*—Drs. J. L. Atlee, of Pennsylvania (Chairman); Horace Carpenter, of Oregon (Secretary).

*Meteorological and Epidemic Diseases.*—Drs. N. S. Davis, of Illinois (Chairman); C. C. Hildreth, of Ohio (Secretary).

*On Medical Jurisprudence.*—Drs. T. Parvin, of Indiana (Chairman); J. A. Murphy, of Ohio (Secretary).

*On Psychology.*—Drs. J. H. Griscom, of New York (chairman); O. F. Renick, of Missouri (Secretary).

Committee of one to assist the Librarian, to reside at Washington, to be continued, Dr. Joseph M. Toner.

S. M. BEMISS,
*Chairman of Committee on Nominations.*

It will be seen, that in addition to the election of the ordinary officers of the Association, officers were also appointed for each section.

This is in compliance with a bye-law, adopted at New

Orleans, in 1869; and was intended to make the sections more permanent and efficient in the accomplishment of the scientific work of the Association. The same bye-law directs, that "papers appropriate to the several sections, in order to secure consideration and action, must be sent to the Secretary of the appropriate section, at least *one month* before the meeting which is to act upon them.

"It shall be the duty of the Secretary, to whom such papers shall be sent, to examine them with care, and, with the advice of the Chairman of his section, to determine the time and order of their presentation, and give due notice of the same; and after their full examination and discussion by the section, they shall be sent to the Permanent-Secretary of the Association."

If this bye-law should be complied with, by those intending present papers, it will enable the officers of each section to place on a card, directly before the Association, at the beginning of each session, the principal work to be done in each section and the time of doing it; which would be a great accommodation to the members, and insure a much more efficient consideration of the papers presented. It is hoped that this arrangement will not be forgotten by all special committees and individuals preparing reports or papers for the Association.

If those connected with the medical press would give some of the space devoted to finding fault with the Association, to notices of these and other arrangements, calculated to promote method and efficiency, in the attention to strictly scientific and practical matters, they would do much more to advance the character and usefulness of our national organization.

---

Chicago Medical College and Female Students.—In compliance with the urgent request of certain parties, the Faculty of the Chicago Medical College last year opened the doors of that Institution for the admission of Female Students; and three or four ladies attended regularly the whole term. They were treated by the young men with respect and propriety, and no serious difficulty arose from their presence. It was true, however, that patients were sometimes found, both in the Hospital and Dispensary, who objected to being presented for clinical instruction before a mixed audience of males and females. And as there are now measures already being taken to establish a College for the special education of women in medicine, having some connection with the Hospital for Women and Children, in this City; and perhaps another in connection with the University of Michigan, at Ann Arbor, the Trustees and Faculty of the Chicago Medical College have declined to receive any more Female Matriculants. Consequently there will be no more mixed classes either in the Chicago Medical College or in the Mercy Hospital.

---

Diagnosis of Renal Disease from Blood-corpuscles in the Urine.—Dr. Joseph G. Richardson, Microscopist to the Pennsylvania Hospital, Philadelphia, has applied to the elucidation of the condition of the kidney in renal disease the well-known views of Cohnheim, regarding the relation of the white blood-corpuscles to the inflammatory process. If both red and white corpuscles are present in the urine, and if it can be ascertained that their occurence is independent of vesical irritation, the following indication may be derived from their relative proportion.

When the red corpuscles greatly exceed the white in number, true hemorrhage has taken place from the kidney. This conclusion will be strengthened if no tube-casts be found, and the albumen be present in such quantity as will correspond to the amount of corpuscles, supposing both to be furnished by the liquor sanguinis.

When the white corpuscles are more than $\frac{1}{25}$th of the red, there is inflammation of the kidney of either an acute or a sub-acute kind, and the danger will be found to be in direct proportion to the total amount of the corpuscles.

When few or no red corpuscles are found to be mixed with the white, chronic inflammation is present, and is proportionate in extent to the number of blood-corpuscles.

In cases of acute Bright's disease, passing into the stage of fatty degeneration, the appearance in the urine of epithelium, containing oil globules points to the change from the stage of inflammation, and these epithelial cells will be found, according to the author, to vary inversely with the number of white corpuscles present, the albumen remaining the same in amount.—*Baltimore Medical Journal.*

### Mortality for the Month of May, 1870.

| | | | | | |
|---|---|---|---|---|---|
| Accident, drowning | 2 | Dysentery | 1 | Neuralgia | 1 |
| " asphyxia | 1 | Dyspepsia | 1 | Old age | 3 |
| " by fall | 3 | Endo-pericarditis | 1 | Paralysis | 2 |
| " gun-shot wound | 1 | Entero-colitis | 1 | Pertyphitis | 1 |
| " railroad | 3 | Enteritis | 5 | Pericarditis | 1 |
| " shock to system | 1 | Epilepsy | 1 | Peritonitis | 3 |
| " suffocation | 1 | Erysipelas | 6 | " puerperal | 1 |
| Anasarca | 2 | Exhaustion, from hem- | | Pleurisy | 1 |
| Anæmia | 1 | orrhage | 1 | Pneumonia | 22 |
| Angina | 1 | Fever, puerperal | 2 | " & complications | 3 |
| Apoplexy | 9 | " remittent | 1 | " typhoid | 1 |
| Asthma | 1 | " scarlet | 20 | Pyelitis, following cys- | |
| Atelectasis pulmonum | 2 | " " complications | 7 | titis | 1 |
| Albuminuria and old- | | " " malignant | 5 | Rheumatism | 1 |
| age | 1 | " typhoid | 8 | " and pericarditis | 1 |
| Births, premature | 18 | Foxemia following | | Scrofula | 2 |
| " still | 39 | childbirth | 1 | Small-Pox | 2 |
| Breast, cancer of | 1 | Gastritis | 2 | Spine disease | 1 |
| Bowels, intussuscept'n | 1 | Hæmoptysis | 1 | " inflammation | 1 |
| Brain, congestion | 5 | Hæmorrhagia, general | 1 | " and brain disease | 1 |
| " inflammation | 5 | Heart, dropsy | 1 | Spina bifida | 1 |
| " ramollissement | 1 | " congestion | 1 | Stomach, cancer | 2 |
| " softening | 3 | " disease | 1 | Strangulation, intern'l | 1 |
| Bronchitis | 4 | " organic disease | 1 | Suicide, by drowning | 3 |
| " chronic | 1 | " valvular disease | 2 | " poison | 2 |
| " capillary | 2 | Hydrocephalus | 8 | " shooting | 3 |
| Cancer of face | 2 | " acute | 3 | Suffocation, by tumor | |
| Chorea and convuls'ns | 1 | Inanition | 14 | of neck | 1 |
| Chlorosis | 1 | Intemperance | 1 | Tabes mesenterica | 16 |
| Cholera Infantum | 6 | Laryngitis | 3 | Teething | 3 |
| Consumption | 57 | Liver, hypertrophy | 1 | " & complications | 3 |
| Convulsions | 50 | Lungs, congestion | 7 | Tetanus | 1 |
| Croup | 9 | " gangrene | 1 | Uræmia | 1 |
| Cynanch tonsillaris | 1 | Manslaughter | 1 | Uterus, cancer of | 2 |
| Debility, general | 3 | Mania, acute | 1 | " hemorrhage | 1 |
| Diarrhœa | 4 | Measles | 5 | Whooping-cough | 6 |
| " chronic | 1 | " & complications | 7 | " & complications | 3 |
| Diphtheria | 7 | Meningitis | 9 | | |
| Dropsy, general | 4 | " cerebro-spinal | 4 | Total | 436 |
| " of chest | 1 | " tubercular | 3 | | |

### COMPARISON.

Deaths in May, 1870, 436 | Deaths in May 1869, 372 | Increase, 64
Deaths in April, 1870, 494 | Decrease, 58

### AGES.

| | | | | | |
|---|---|---|---|---|---|
| Under 1 | 138 | 10 to 20 | 24 | 60 to 70 | 10 |
| 1 to 2 | 56 | 20 to 30 | 37 | 70 to 80 | 5 |
| 2 to 3 | 21 | 30 to 40 | 45 | 80 to 90 | 1 |
| 3 to 4 | 14 | 40 to 50 | 34 | | |
| 4 to 5 | 6 | 50 to 60 | 17 | Total, | 436 |
| 5 to 10 | 28 | | | | |

Males, 235 | Females, 201 | Total, 436
Single, 329 | Married 107 | Total, 436
White, 431 | Colored, 5 | Total, 436

NATIVITY.

| | | | | | |
|---|---|---|---|---|---|
| Austria | 1 | England | 13 | Norway | 10 |
| Bohemia | 1 | France | 2 | Sweden | 7 |
| Canada | 7 | Germany | 44 | Scotland, | 6 |
| Chicago, Native | 64 | Holland | 3 | Unknown | 2 |
| Chicago, Foreign | 166 | Ireland | 46 | | |
| U. S., other parts | 62 | New Brunswick | 1 | Total, | 436 |
| Denmark | 1 | | | | |

MORTALITY BY WARDS FOR THE MONTH.

| Wards. | Mortality. | Wards. | Mortality. |
|---|---|---|---|
| 1 | 8 | 19 | 14 |
| 2 | 10 | 20 | 20 |
| 3 | 21 | Accidents | 12 |
| 4 | 5 | County Hospital | 6 |
| 5 | 9 | Home for Friendless | 2 |
| 6 | 19 | Hospital Alexian Brothers | 1 |
| 7 | 25 | Immigrants | 9 |
| 8 | 40 | Jewish Hospital | 1 |
| 9 | 36 | Lake Hospital | 1 |
| 10 | 10 | Marine Hospital | 1 |
| 11 | 17 | Mercy Hospital | 4 |
| 12 | 20 | Manslaughter | 1 |
| 13 | 9 | St. Luke's Hospital | 1 |
| 14 | 12 | St. Joseph Orphan Asylum | 14 |
| 15 | 29 | Suicide | 8 |
| 16 | 23 | | |
| 17 | 25 | Total, | 436 |
| 18 | 23 | | |

Glycogenic Function of the Liver.—Dr. Austin Flint, Jr. (Glycogenic Function of the Liver. New York Medical Journal, January, 1869), removed in a number of cases a portion of the liver from a living animal, and having plunged it into boiling water to arrest the fermentation of its glycogenic matter, demonstrated in it the absence of sugar. Then killing the dog, and rapidly tying the portal vein, and the vena cava above and below the hepatic veins, demonstrated in blood from the latter vessels an abundant presence of sugar. Whence he concluded that during life the glycogenic matter is undergoing continual change into sugar, but is not found in the liver-tissue proper, because it is washed out by the blood as soon as it is formed.

Pavy, in the September number of the same Journal, replies that the delay in applying ligatures to the vessels after destroying life is sufficient for a *post mortem* production of sugar to take place, and that the sugar found in the hepatic veins by Dr. Flint was, consequently, the product of *post mortem* fermentation. (It can, however, be conclusively demonstrated that the quantity of sugar taken from the right side of the heart, though in itself trifling, is really much larger than what is found in vessels of the body more remote from the liver.—W. T. L.)—*New York Medical Journal.*

---

THE PHYSIOLOGY OF THE PANCREATIC SECRETION.—(N. O. Bernstein. Central Blatt., No. xlv.) Bernstein established permanent pancreatic fistulæ in animals by introducing into the pancreatic duct a lead wire, the two extremities of which were inserted into the opening while the central portion was so twisted as to form of the whole a letter T. The wire was thus retained it the duct without a ligature. The secretion was caught by a funnel covering the wound, and terminating in a graduated tube. The fluid obtained continued to present the digestive properties of the pancreas. While fasting no secretion took place. The latter began during the first hour after food had been taken, and reached its maximum in the second or third hour, then fell, to increase again between the fifth and seventh hour, then again sank, and finally disappeared in about fifteen hours. There was complete suppression during vomiting. Division of the vagus produced no effect, while irritation of the centric end was followed by suppression. Division of the pancreatic nerves proper, which accompany the arteries, produced a continued and very abundant secretion of normal juice, and the gland became red and œdematous. Woorari poisoning produced an increased secretion.—*New York Medical Journal.*

---

CONSTITUENTS OF ERGOT.—Although it is some years since the discovery of two alkaloids existing in ergot of rye, the fact is, as yet, little known to the profession. A recent confirmation of the discovery, brings the matter again to notice, and this, together with the statement, in a translation by Dr. Deane, of an article on the hypodermic use of ergot, as published in the *California Medical Gazette*, that the chemical property of ergot which produces the specific result has not been determined, renders the following account of interest. In 1864, Mr. Wenzell, then of La Crosse, Wisconsin, but now permanently located in San Francisco, published in the *American Journal of Pharmacy* the results of an analysis of ergot, stating that he had succeeded in isolating two new alkaloids, *ecbolin* and *ergotin*, and an organic acid, ergotic acid, besides trymethylamin, (pseudopropylamin) previously discovered by Winkler. The existence of these, Mr Wenzell established to his own satisfaction by means of qualitative reactions, as newly discovered and not previously described. Subsequently, Mr Manassewitz repeated Mr Wenzell's experiments and obtained ergotin, but failed to isolate the acid and the *ecbolin.* In 1869, however, J. Carl Hermann, (*Wittstein's Viertelj. Schr.*) succeeded in isolating the *ecbolin*, thus fully confirming Wenzell's discovery. It remains to be ascertained whether the active principles of the drug reside in the alkaloids. Mr Wenzell informs us that in some experiments directed to this end he obtained from the alkaloids all the effects usually produced by ergot.

---

Tetanus Produced by the Administration of Quinia Hypodermically.—A case of this character is reported by E. Paul Sale, M.D., Aberdeen, Miss., in the *N. O. Jour. of Medicine.* The patient, aged nineteen, mother of twins, æt. three months, was suffering from *malarial coma*, the result of a tertian intermittent fever of two months' duration. Desiring to rapidly quininize her, he administered in the arm, by hypodermic injection, quinia, gr. vj., of an ethereal solution.

Four days afterwards the arm was much tumefied, hot, and very painful to the touch, when the syringe was inserted. Twelve days from the injection, Dr. Sale was summoned to the patient's bedside to prescribe for trismus with opisthotonos; but she succumbed after receiving the best of treatment. The chief point developed by this case is this: It shows the deleterious effects which frequently follow the use of quinine hypodermically. This is the fourth case out of ten in which he has had cause to regret resorting to this method of medication, on account of the violent inflammation which has been the sequence.

---

The Administration of Chloral.—J. M. Da Costa, M.D. (*Am. Jour. Med. Sciences*), in his "Clinical Notes on Chloral," thus observes: With reference to the action of chloral on coughs it is sufficient. Nor did it do any good in the only case of asthma in which he tried it. He has also been disappointed in its use in hiccough and in chorea. It is important to know that this powerful agent is not devoid of danger, and in every case we should administer it in tentative doses and carefully note its effects. It is contraindicated in some diseases of the heart, and ought not to be prescribed for patients with feeble heart. Some of the unpleasant effects of the drug can be avoided by the simultaneous administration of opium. The two remedies can be used advantageously in some cases of delirium tremens, and when we wish to quiet pain. In conclusion, he believes that it is an important addition to our therapeutic means—chiefly, however, as a hypnotic. Its action as a destroyer of pain is limited, and it cannot compete with ether or chloroform in producing the insensibility requisite for surgical operations. Its chief value seems to be that of an auxiliary to opium, or to take its place when opium is not admissible. It is, however, not so certain; nor is it likely to displace that peerless drug.—*Medical Record.*

---

Ovariotomy Twice on One Patient.—M. Boinet exhibited to the Paris Academy of Medicine (*Lancet*) a woman of 48, in good condition, from whom he had removed both ovaries. The left ovary, first-removed, weighed 36 pounds, and the other, removed ten years afterwards, 18 pounds. At the second operation, she was very near dying from diphtheria, which was limited to the air-passages, not affecting the wound. M. B. always includes the peritoneal edge in the sutures of the lips of the wound.

---

Shock.—Prof. J. T. Hodgen, St. Louis, in a recent paper on fractures, says he cannot imagine a case of shock in which stimulation would save a life that would be lost without it. He advises quiet, exclusion of light and sound, and after a few hours, if the patient calls for it, gives milk, or essence of beef, but no *stimulant as such* under any circumstances.—*St. Louis Medical and Surgical Journal.*

---

Ergot in Hemoptysis.—Dr. Drasche states that he has used ergot hypodermically in hemoptysis with entire success. Von Graeffe has gotten similar results. Dr. Plagge thinks that ergot, administered subcutaneously, is the most reliable agent in this affection, arresting the hemorrhage almost instantaneously by inducing coagulation of the blood.—*Dr. Henry, in Cincinnati Medical Repertory.*

---

Morphia as the Test for Opium.—It has been generally regarded that the quality of opium may be determined by the proportion of morphia it contains. But owing to value of the other active principles contained by it, and which may not exist in the same proportions as morphia, pharmacists are tending to the opinion that the morphia test is not altogether correct.

---

Bromide of Potassium in Saccharine Diabetes.—Prof. Austin Flint, Sr., reports in the *American Practitioner*, three cases of saccharine diabetes in which he prescribed the bromide of potassium in 15 to 20 grain doses three times daily, with ordinary anti-diabetic diet, with very marked benefit. There was in each a rapid diminution of the thirst, a decrease of the amount and specific gravity of the urine to about a normal condition, and a corresponding improvement in the general health and condition of the patients.—*Medical Record.*

---

A Chair of the History of Medicine. The late M. Salmon de Champotran, of the Council of State, Paris, left to the Faculty of Medicine of Paris a legacy to found a chair of the history of medicine. M. Bouchut is the prominent candidate.

---

Cinchona.—The culture of the cinchona, or Peruvian bark, in St. Helena is progressing. The plants all look flourishing. There are now about 4000 of them.

---

Medical "Wrinkles."—The quaint and practical Thomas Inman, M.D., of Liverpool, in one of his readable essays on the "Restoration of Health," thus remarks: "Do you wish to ascertain the health of a baby, feel the condition of its buttocks. If these are firm and elastic, one may always be sure that the little one is strong and well; but if, on the other hand, they are soft, as if they were boiled turnips in a bladder, it is certain that the child is out of sorts.

---

Treatment of Epilepsy.—George Johnson, M.D., F.R.C., Physician to King's College Hospital, advocates chloroform in connection with bromide of potassium in this affection. Chloroform wards off a threatened fit, and cuts short a violent and prolonged paroxysm.

